# Treatment and Outcome for Children with Esophageal Atresia from a Gender Perspective

**DOI:** 10.1155/2017/8345798

**Published:** 2017-10-22

**Authors:** Julia Ekselius, Martin Salö, Einar Arnbjörnsson, Pernilla Stenström

**Affiliations:** Department of Pediatric Surgery, Lund University and Skåne University Hospital, Lund, Sweden

## Abstract

**Background:**

Besides the incidence of esophageal atresia (EA) being higher in males, no other gender-specific differences in EA have been reported. The aim of this study was to search for gender-specific differences in EA.

**Methods:**

A retrospective study was conducted at a tertiary center for pediatric surgery. The medical charts of infants born with EA were reviewed. 20 girls were identified, and 20 boys were selected as matched controls with respect to concomitant malformations. Their treatment and outcome were evaluated.

**Results:**

Polyhydramnios was more common in pregnancies with boys, 40%, versus girls, 10%, with EA (*p* < 0.01). In total, 36 (90%) children had patent ductus arteriosus, without any gender difference (18 and 18, resp., *p*=1). The distribution of days at the different levels of care was not equally distributed between boys and girls. Boys with EA had significantly more postoperative days (median 5 days) in the ward than girls (median 5 and 2 days, resp., *p*=0.04). No other gender-specific differences in surgical treatment, complications, or symptoms at follow-up were identified.

**Conclusion:**

Polyhydramnios appears to be more frequent in pregnancies with boys than girls with EA. In this study, boys have longer stays than girls at the pediatric surgery ward.

## 1. Introduction

Esophageal atresia (EA) is reported to be more common among boys than girls [1–3]. However, it is never reported on whether gender in praxis affects the care or outcome of patients with EA. Therefore, it is important to assess a cohort of children with EA with respect to gender, to secure equal treatment and adequate care for all. The aim of this study was therefore to investigate if there are any gender-specific differences in children with EA.

## 2. Patients and Methods

### 2.1. Study Design

The study was a descriptive retrospective study conducted at a tertiary center for pediatric surgery. The medical charts of all children born with EA between January 2001 and February 2016 were reviewed. Data were collected with focus on potential gender differences with regard to (1) birth details, (2) surgical course and postoperative intervention, (3) postoperative complications, and (4) physical symptoms during the first year. The review was carried out by one independent researcher without previous connection to the patients included in the study. Each child was followed for one year, the endpoint of the study.

### 2.2. Routines and Definitions of Terms

#### 2.2.1. Local Routines of Care

The postoperative care during the first day or days after esophageal reconstructive surgery is at any of the two intensive care units–the neonatal (NICU) or pediatric intensive care unit (ICU). Then, the care is continued at the intermediate intensive care unit and then at the ward of pediatric surgery, before discharge. The discharge from the hospital could be either directly to home or to a local hospital.

#### 2.2.2. Definitions

Dysphagia was defined as any reported difficulty to swallow food or liquid, resulting in prolonged time and effort to finish a meal. Gastroesophageal reflux (GER) was defined to include symptoms such as belching, vomiting, frequent burping, and refusal to eat, or trouble eating. Both dysphagia and GER were parental reported.

Congenital cardiac shunts were defined as heart defects at birth, including patent ductus arteriosus, patent foramen ovale, ventricular septal defects, and atrial septal defects. These defects were only considered to be cardiac malformations when they had a hemodynamic effect. Central venous catheter (CVC) infection was registered when treated with antibiotics. Change of drain was registered when a new drain replaced the thorax drain postoperatively, contrary to extra drain which was registered when an additional drain was received postoperatively.

### 2.3. Patients

The inclusion criterion for the study was children with EA with a distal tracheoesophageal fistula (TEF) (Gross type C). Excluded were children with other types of EA, children who had undergone surgery at another hospital, and children from other centers who had their esophageal reconstruction at the hospital because of concomitant cardiac malformations but later were followed at their local hospital. All girls who met these criteria were included in the study. One boy was selected as a matched control for each girl. The selection of boys was based on associated malformations only, with the intention to make the two gender groups as much alike as possible from a medical perspective. Thus, the female group was a cohort while the selection for pairing was performed in the male group.

### 2.4. Method of Operation

All children included were operated on with the same operation technique that was unchanged over the study period. The operation was performed through a posterolateral extrapleural right-sided thoracotomy. The azygous vein was exposed and ligated. The TEF was identified and closed with absorbable stitches. The upper esophageal pouch was mobilized from the proximal trachea. Anastomosis with single-layer 5/0 sutures was performed, and a trans-anastomotic feeding tube (6–8 French) was used for postoperative feeding.

### 2.5. ICU/NICU Strategies

According to the ICU/NICU strategies, the children stayed in ICU/NICU as long as they needed respiratory and/or circulatory support. This strategy did not change over the study period.

### 2.6. Statistical Analysis

Power calculations were performed using PS Power and Sample Size Calculation v.3.0 (http://biostat.mc.vanderbilt.edu/wiki/Main/PowerSampleSize) [4]. The following background for power calculation was calculated on by a statistician: clinically significant relevance for the examined parameters was considered as a difference of 30%. The study matched case and control sets (1 matched control per case). Preliminary data indicated that the probability of exposure among controls was 0.5, and the correlation coefficient for exposure between matched cases and controls was 0.1. If the true odds ratio for positive finding in exposed subjects with respect to unexposed subjects was 0.1, then we would need to examine 19 case patients with 1 matched control per case to reject the null hypothesis that the odds ratio equals 1 with probability (power) of 0.8. The type I error probability associated with the test of this null hypothesis is 0.05.

All data were compiled using Microsoft Excel, and all statistical analyses were performed using the Social Science Statistics site (http://www.socscistatistics.com) [5]. Fisher's two-tailed exact test was used for comparison of dichotomous data, and the Mann–Whitney *U* test was used for comparison of continuous data, to compute statistical significance. A *p* value < 0.05 was considered statistically significant.

### 2.7. Ethical Considerations

This study was executed according to the Helsinki Declaration and approved by the Regional Ethical Review Board (registration number 2010/49). Data were anonymized before analyses, and the presentation method precludes participant identification. The study did not affect the care given to each patient. All patients were treated per the standard care for patients with EA at the pediatric surgical center and according to national guidelines.

## 3. Results

During the selected study period of 15 years, there were a total of 77 live births of infants with EA in the region corresponding to 5 newborns each year with an insignificant slight increase in the number of patients yearly during the period studied. The mortality was zero. The female : male ratio was 1 : 4 during this period.

In total, seven (five boys and two girls) were excluded according to the exclusion criteria: patients with Gross type A (*n* = 3) and Gross type B (*n* = 1) and those who belonged to other hospitals (*n* = 3). The medical charts of the remaining 70 patients were reviewed. Out of these patients, 20 were girls. All girls were included in the study and matched with 20 boys with respect to concomitant malformations.

### 3.1. Birth Parameters and Concomitant Malformations

Polyhydramnios was present in 25% of all pregnancies and turned out to be more common in pregnancies with boys (40%) than girls (10%) with EA (*p* < 0.01). No gender-specific differences were detected with respect to birth weight, gestation age, or prenatal suspected diagnosis (Table 1). One or more concomitant malformations were present in 35% of the girls and in 30% of the boys. The most common abnormality was vertebral malformation, which was present in 25% of girls with EA. All children had cardiac examinations performed before surgery. Patent ductus arteriosus was identified in 18 girls and 18 boys, corresponding to a total of 90%. Manifest cardiac malformations with hemodynamic influences were found in four patients, and three of them had cardiac surgery and one was planned for it at the time of the study (Table 2). No children had any detected chromosomal abnormalities.

### 3.2. Medical Care and Treatment

The time to surgery from birth did not significantly differ between girls and boys with EA (median 1 day for both genders), nor did the percentage of patients receiving oral feeding before diagnosis of EA (25% and 35%, resp.). Furthermore, no significant differences in frequencies of thorax drain placement during surgery or postoperative complications within 30 days were detected (Table 3). The time until the first tube feeding was median 2 days for both girls and boys, and the time until oral feeding was median 9 days for girls and 10 days for boys, without any statistical difference. Frequency, as well as type of postoperative complications, did not differ between boys and girls. In analyses regarding need of postoperative care at different care levels (children intensive care, neonatal intensive care, and pediatric surgery ward), significantly longer postoperative stays at the pediatric surgery ward were registered for boys than girls with EA, while there was no significant difference in time needed at the intensive care departments or in the total stay (Table 4).

### 3.3. Outcome at Three-Month Control and at One-Year Follow-Up

**Figure 1:**
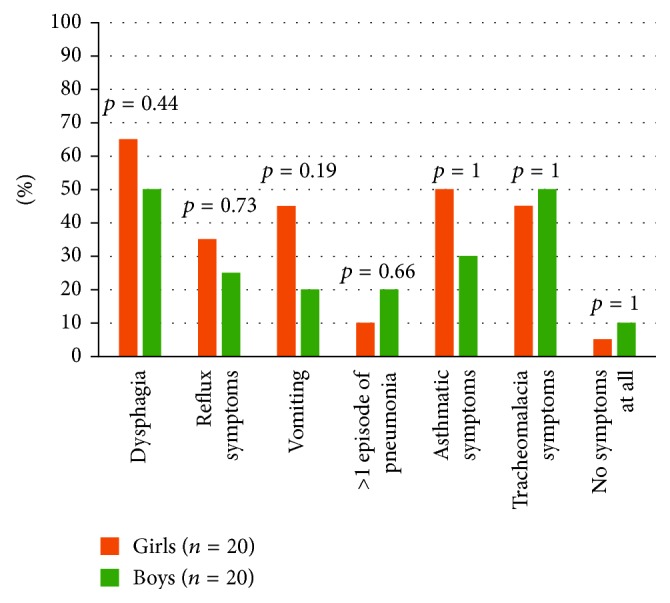
Gender comparison of parental-reported symptoms in children with esophageal atresia at the 1-year control after reconstruction. *n* = number. Statistical method: Fisher's two-tailed exact test.

In the three-month control, there were no gender-specific differences in anastomotic stricture or prestenosis dilatation, or any difference in reported symptoms (Table 5). During the whole first year, there were no gender-specific differences in the time to first dilatation of the anastomotic strictures in esophagus, or in dilatation frequencies (Table 6). The one-year follow-up still included 20 boys and 20 girls with EA. One girl had surgery during her first year of life due to severe tracheomalacia. Also during the first year, one girl (out of two) and two boys (out of two) with associated heart conditions had cardiac surgery. The most frequently reported symptoms during the year after surgery were dysphagia, vomiting, and asthma/coughing for girls and dysphagia and tracheomalacia for boys (Figure 1()).

## 4. Discussion

The results of this study indicate that polyhydramnios was more common in pregnancies with boys than girls with EA, and the distribution of days spent at different care levels after reconstruction turned out to be skewed since the boys had significantly more days in the pediatric surgery ward. No other gender-specific differences were detected for patient characteristics, care, or outcome.

The finding that polyhydramnios was more common in pregnancies with boys than girls with EA has not, to the authors' best knowledge, been assessed or reported in any previous study. However, the overall prevalence of polyhydramnios was 25% in our cohort, which is low compared to previous studies where polyhydramnios in EA pregnancies has been reported to be 55% without any gender analysis made [6, 7]. One reason for differences in prevalence could be that polyhydramnios is defined differently or registered differently with regard to pregnancy length or by different routines. Also, polyhydramnios is not specifically correlated with EA; it also occurs as an idiopathic condition [8]. The gender distribution in studies reporting on idiopathic polyhydramnios reports a gender distribution of 7 boys : 3 girls [9], which is in line with our results. Polyhydramnios could also be associated with other malformations, and in our study, the frequency of concomitant malformations (25%) is lower than in some previous reports on EA (50–55%) [1, 6, 7]. This may be reflected in the lower frequency of polyhydramnios found in our study.

The reason for the gender difference in the frequency of polyhydramnios is not clear and cannot be explained by any physiological factors related to EA. The finding cannot be related to any other pathology prenatally diagnosed, since this has not been studied before by our group. In a recent publication about idiopathic polyhydramnios, a similar gender difference was found [9]. Thus, it seems that EA on its own has no significance and impact for gender differences in polyhydramnios, and our finding can probably not be defined as specific for this selected group of patients. This is especially with regard to the limited numbers of children included in our study.

At our hospital, boys with EA spent a longer time in the pediatric surgery ward after surgical reconstruction and before discharge than the girls did. The question could be raised, if the result mirrors a true gender difference in care or if it is caused by that several care levels are involved in the analysis which might blur the picture. The total number of days spent in the hospital did not differ between the genders, nor did the time at any of the intensive care units, but the girls were discharged from the surgical ward earlier than the boys. Although there was a lack of significant gender differences in birth weight and SGA, it cannot be excluded that those parameters still might influence the time in the ward. However, it might also be explained by geographical reasons such as if the girls came from places closer to the hospital. Then, days at the ward might have been substituted with extra controls in the outpatient clinic after discharge. However, this parameter was not included in analyses. In a previous study, our group presented gender analysis on children with Hirschsprung disease [10]. In that study, the length of hospital stays did not differ between girls and boys, but the boys had more emergency admissions during the first postoperative year.

Some results provide novel insights and raise new questions regarding children born with EA and concomitant cardiac findings. A great majority (90%) of the included children had patent ductus arteriosus. We did not detect significant gender-specific differences with respect to the incidence of these heart defects, but the frequency overall appears to be higher in children with EA. Previous research have suggested that patent ductus arteriosus and patent foramen ovale are present in 45% and 62% of full-term babies during their first 60 hours of life [11]. The finding is therefore going to be further studied.

The strength of the study was that all included patients had followed the same standard of care per the local strict care and follow-up programs. The staff at the center routinely conducted detailed and continual documentation in the medical records, which made it possible to collect essentially all needed data, although it was a retrospective study. Another strength was that the two groups could be considered comparable out from a balance of other concomitant malformations, which otherwise could have influenced physical outcome and/or time of care. The obvious limitations of the study are the retrospective design and small numbers of patients. Thus, the results only present a hypothesis.

Also, it cannot be excluded that the information about polyhydramnios may be weak, since it is based on the information documented in the children's medical charts, and not the mothers', although per the local routines, this should be documented correctly also for the child. The small study population reduces the chance to prove any significance and increases the risk of making a type II error. Therefore, one should be aware that the lack of significance does not mean gender equality or that the genders were equally treated.

In previous studies on EA, several evaluations similar to ours have been made, but none have presented gender analyses in EA. No significant and important gender-specific differences have been detected and caused important changes in our clinical routines. It is important to consider that both biological and social differences in girls and boys, or attitudes among care personnel, could constitute potential determinants of outcome also in pediatric surgery, and therefore gender aspects should be enlightened so that the care can be improved in the best way for all children.

## 5. Conclusion

The results of this study suggest that polyhydramnios is more frequent in pregnancies with boys than girls with EA. In the study, boys spend more days in the pediatric surgery ward than girls, before being discharged, which cannot be explained by differences in birth characteristics or concomitant malformations.

It is important to continue research and investigating gender-specific aspects of EA and to perform future studies with larger patient cohorts. This is since gender analysis of biological parameters in EA, medical care, and outcome will help optimizing care and the final outcome for all children born with EA.

## Figures and Tables

**Table 1:** Comparison of birth parameters of girls and boys born with esophageal atresia (EA). Values are presented as median (range) or as the number and percentage of patients, *n* (%). SGA: small for gestational age.

	Girls (*n* = 20)	Boys (*n* = 20)	*p* value
Gestational age, weeks + days median (range)	39 + 2 ((30 + 5)–(42 + 5))	39 + 3 ((34 + 2)–(42 + 2))	0.87^∗^
Birth weight, gram median (range)	2933 (1410–4065)	3215 (1480–4400)	0.23^∗^
SGA, *n* (%)	5 (25)	1 (5)	0.18^∗∗^
Polyhydramnios, *n* (%)	2 (10)	8 (40)	0.04^∗∗^
Prenatal suspicion of EA, *n* (%)	1 (5)	0 (0)	1^∗∗^

^∗^Mann–Whitney *U* test; ^∗∗^Fisher's exact test.

**Table 2:** Concomitant malformations of the included girls and boys with esophageal atresia. Values are presented as number and percentage of patients, *n* (%). Genital malformations were not included.

	Girls (*n* = 20)	Boys (*n* = 20)	*p* value^∗^
V (vertebral malformation)	5 (25)	2 (10)	0.41
A (anal malformation)	2 (10)	2 (10)	1
C (cardiac malformation^∗∗^)	2 (10)	2 (10)	1
T (tracheoesophageal fistula)	20 (100)	20 (100)	1
E (esophageal atresia)	20 (100)	20 (100)	1
R (renal malformation)	1 (5)	1 (5)	1
L (limb malformation)	1 (5)	1 (5)	1
Duodenal malformation	1 (5)	2 (10)	1
Spinal cord malformation	1 (5)	0 (0)	1
Cleft lip and palate malformation	1 (5)	0 (0)	1
Congenital cardiac shunts	18 (90)	18 (90)	1

^∗^Fisher's exact test; ^∗∗^cardiac malformations were here defined as malformations with hemodynamic influence. Three out of the four children were operated on, and a cardiac surgery was planned for the fourth patient.

**Table 3:** Gender comparison of preoperative treatment, postoperative treatment, and complications within 30 days after reconstruction of esophageal atresia. Values are presented as median (range) and number and percentage of patients, *n* (%).

	Girls (*n* = 20)	Boys (*n* = 20)	*p* value
	Median (range)	Median (range)	
*Preoperative treatment*			
Days until surgery, median (range)	1 (0–2)	1 (0–3)	0.15^∗^
Received food before surgery	5 (25)	7 (35)	0.73^∗∗^
*Surgical treatment*			
Received CVC	13 (65)	15 (75)	0.73^∗∗^
Received thoracic drain	16 (80)	17 (85)	0.66^∗∗^
*Postoperative treatment with drain*			
Change of thoracic drain	2 (10)	0 (0)	0.49^∗∗^
Extra thoracic drain	6 (30)	4 (20)	0.72^∗∗^
Insertion of drain if not placed at surgery	0 (0)	1 (5)	1^∗∗^
*Complications*			
Anastomotic leakage	3 (15)	4 (20)	1^∗∗^
Pneumothorax	10 (50)	11 (55)	1^∗∗^
Treated sepsis	3 (15)	4 (20)	1^∗∗^
Treated wound infection	3 (15)	6 (30)	0.45^∗∗^
Treated CVC^∗∗∗^ infection	1 (5)	0 (0)	1^∗∗^

^∗^Mann–Whitney *U* test; ^∗∗^Fisher's exact test; ^∗∗∗^CVC: central venous catheter.

**Table 4:** Gender comparisons of postoperative details and care after reconstructive esophageal surgery, including number of days spent at different departments. Values are presented as median (range) or number and percentage of patients, *n* (%). The intensive care units were pediatric intensive care unit and neonatal intensive care unit.

	Girls (*n* = 20)	Boys (*n* = 20)	*p* value
*Postoperative treatment*			
Days until first tube feeding	2 (1–6)	2 (1–32)	0.76^∗^
Days until postoperative radiological contrast examination of esophagus	9 (3–29)	8 (4–13)	0.27^∗^
Days until first per oral feeding	10 (5–36)	9 (6–41)	0.60^∗^
*Care level*			
Postoperative days in any intensive care unit	0 (0–31)	0 (0–22)	0.21^∗^
Postoperative days in the intermediate intensive care unit	8 (0–26)	6 (1–31)	0.71^∗^
Postoperative days in the pediatric surgery ward	2 (0–10)	5 (0–22)	0.04^∗^
Total stay (days)	22 (9–58)	14 (8–84)	0.04^∗^
*Discharged*			
Discharged to home, *n* (%)	11 (55)	14 (70)	0.51^∗∗^
Discharged to a local hospital, *n* (%)	9 (45)	6 (30)	0.51^∗∗^

^∗^Mann–Whitney *U* test; ^∗∗^Fischer's exact test.

**Table 5:** Details regarding examination and findings at 3-month control, including esophageal X-ray and parental reports of symptoms indicating anastomotic stricture. Values are presented as median (range) or number and percentage of patients, *n* (%).

	Girls (*n* = 20)	Boys (*n* = 20)	*p* value
	Median (range)	Median (range)	
Weeks to contrast X-ray after discharge, median (range)	6 (1–11)	9 (1–15)	0.07^∗^
*Findings at X-ray*			
Anastomotic stricture, *n* (%)	13 (65)	12 (60)	0.74^∗∗^
Prestenosis dilatation, *n* (%)	10 (50)	11 (55)	0.75^∗∗^
*Parental-reported symptoms*			
Symptoms of anastomotic stricture, *n* (%)	11 (55)	12 (60)	1^∗∗^
*Objective findings*			
Findings leading to examination under anesthesia, *n* (%)	13 (65)	14 (70)	1^∗∗^
Findings under anesthesia leading to dilatation, *n* (%)	9 (45)	10 (50)	1^∗∗^

^∗^Mann–Whitney *U* test; ^∗∗^Fisher's exact test.

**Table 6:** Need of dilatations and anesthesia for anastomotic stricture among boys and girls with esophageal atresia during the first year after surgery. Values are presented as median (range) or as the absolute number and percentage of patients, *n* (%).

	Girls (*n* = 20)	Boys (*n* = 20)	*p* value
	Median (range)	Median (range)	
≥1 dilatation	*n* = 12 (60%)	*n* = 12 (60%)	1^∗^
Weeks until 1st dilatation	8 (4–49)	11 (4–36)	1^∗∗^
Number of dilatations	1 (0–15)	2 (0–10)	0.24^∗∗^
Number of anesthesia procedures	4 (1–16)	6 (1–14)	0.48^∗∗^

^∗^Fisher's exact test; ^∗∗^Mann–Whitney *U* test.
